# Baseline Correction for NMR Spectroscopic Metabolomics Data Analysis

**DOI:** 10.1186/1471-2105-9-324

**Published:** 2008-07-29

**Authors:** Yuanxin Xi, David M Rocke

**Affiliations:** 1 Department of Applied Science, University of California, Davis, One Shields Avenue, Davis, CA 95616, USA; 2 Division of Biostatistics, School of Medicine, University of California, Davis, One Shields Avenue, Davis, CA 95616, USA

## Abstract

**Background:**

We propose a statistically principled baseline correction method, derived from a parametric smoothing model. It uses a score function to describe the key features of baseline distortion and constructs an optimal baseline curve to maximize it. The parameters are determined automatically by using LOWESS (locally weighted scatterplot smoothing) regression to estimate the noise variance.

**Results:**

We tested this method on 1D NMR spectra with different forms of baseline distortions, and demonstrated that it is effective for both regular 1D NMR spectra and metabolomics spectra with over-crowded peaks.

**Conclusion:**

Compared with the automatic baseline correction function in XWINNMR 3.5, the penalized smoothing method provides more accurate baseline correction for high-signal density metabolomics spectra.

## Background

Baseline distortions in 1D NMR spectra are mainly caused by the corruption of the first few data points in FID (free induction decay). These corrupted data points add low frequency modulations in the Fourier-transformed spectrum, and thus formed the distorted baseline. Correction of these distortions is a necessary step in NMR spectra data processing because they offset the intensity values and result in inaccuracy in peak assignment and quantification. These errors could be critical in the study of metabolomics, which involves many small but statistically significant peaks that are sensitive to baseline distortions. Incorrect quantification of these peaks may result in failures in detection of important metabolites or identifying potential biomarkers.

Existing automatic baseline distortion correction methods fall into two categories: time domain correction and frequency domain correction [1-9]. Time domain correction methods reconstruct the corrupted data points in FID to reduce the low frequency modulation [6-8]. Frequency domain correction methods construct baseline curves in the spectra directly, and subtract these baseline curves to remove the distortion [1-5]. These methods have been implemented in commercial software and hand-written programs for NMR data processing. Usually a combination of both time and frequency domain methods are applied to achieve better correction. In comparison, time domain methods provide general correction for FID and frequency domain methods provide more detailed correction on the spectrum itself. For processing a specific kind of NMR spectra, such as metabolomics spectra, frequency domain methods are easier to tailor for different needs.

In this article, we present a novel frequency domain baseline correction method for processing 1D spectra for metabolomics profiling. 1D proton NMR spectroscopy has been widely applied in metabolomics profiling because it can simultaneously measure many metabolites that lie above the detection limit. These metabolomics spectra usually have many crowded peaks such that noise regions are broken into smaller pieces and are more difficult to identify accurately. The term noise regions refer to the regions in the spectrum that only contain noise. In some part of the spectra, severely overlapping peaks form long gaps between noise regions and are a cause of numerical sensitivity in baseline construction. Traditional frequency domain methods detect noise regions and construct the baseline curve by interpolating among identified noise regions. These methods rely heavily on robust noise region identification, and therefore may not achieve optimal baseline correction in metabolomics spectra. For example, Golotvin et al [5] proposed identifying noise points by comparing the intensity range of a small neighborhood with the standard deviation of noise regions, which is estimated by dividing the spectrum into 32 sections and taking the minimum value of standard deviations of these sections. We observed that this method occasionally identifies the low signal points in metabolomics spectra as noise because they may overlap with each other and have reduced standard deviation, and as a result these signal points will be offset to zero after baseline correction. Noise standard deviation estimation is also theoretically biased to be smaller than the true value in a statistical view, and leads to additional inaccuracy in detection of noise data points.

As an alternative to the existing noise detection and interpolation approaches, we developed a new baseline correction method based on a penalized parametric smoothing model. This method fits a curve following the bottom envelope of the spectrum and doesn't need explicit identification of the noise data points. The primary motivation is that we model the baseline as a smooth curve of arbitrary form that goes through the noise region instead of linked pieces of selected noise points. We describe key features of this model by a score function and construct the optimal baseline curve corresponding to the function maximum. In addition, we present a more accurate estimation of noise variance by LOWESS (locally weighted scatterplot smoothing) regression and use it to determine the model parameters.

## Methods

Suppose a 1D NMR spectrum is represented by the set of ordered pairs {*x*_*i*_, *y*_*i*_}, where *x*_*i *_is the abscissa in ppm units and *y*_*i *_is the ordinate, representing the height of the spectrum as a Fourier-transformed RF decay curve. The fundamental model behind our method is that the spectrum can be represented as

(1) $$y_{i}=b_{i}+\mu_{i}e^{\eta_{i}}+\varepsilon_{i},$$

where *b*_*i *_is the baseline, *μ*_i _is the true signal, and *η*_i _and *ε*_i _are random errors normally distributed with mean 0 and variance 1, generally autocorrelated, a type of model that fits a wide variety of measurement data (Rocke and Lorenzato 1995 [10]; Rocke and Durbin 2001 [11]). An estimated baseline should be 1) smooth, but not necessarily flat; and 2) run through the middle of the data in segments where there is no signal. Based the on these features, we construct the following score function:

(2) $$F(b)=\sum_{i}b_{i}-A\sum_{i}{\left(b_{i+1}+b_{i-1}-2b_{i}\right)}^{2}-B\sum_{i}{\left(b_{i}-y_{i}\right)}^{2}g(b_{i}-y_{i})$$

And $g(b_{i}-y_{i})=\{\begin{array}{cc} 1, & b_{i}-y_{i}>0\\ 0, & b_{i}-y_{i}\leq 0 \end{array}$ is the Heaviside step function.

**b **= {*b*_*i*_} is a set of points that represents a certain baseline. The optimal baseline curve **b**_0 _should maximize the score function *F*(**b**).

(3) **b**_0 _= arg max *F*(**b**)

*F*(**b**) has three components. The first term $\sum_{i}b_{i}$ is the sum of all baseline points. We want to maximize it subject to the smoothness penalty $-A\sum_{i}{\left(b_{i+1}+b_{i-1}-2b_{i}\right)}^{2}$ and the negativity penalty $-B\sum_{i}{\left(b_{i}-y_{i}\right)}^{2}g(b_{i}-y_{i})$. The smoothness penalty is a discrete form of integral of squares of second-order derivatives, which is small for linear segments and large for small curvature radius. The negativity penalty is designed to be nonzero only when the baseline point is above the data point, by using the Heaviside step function *g*(*b*_*i *_- *y*_*i*_). It counteracts the uptrend of the first term and force the baseline to run through the middle of the data. By maximizing this function the baseline is pushed up to the spectrum but not exceeding the zero-signal level, and forced to be as smooth as possible to link peak regions.

The negative penalty parameter *B *is determined by the condition that the baseline should run through the center of the noise region. Take the simplest case of a spectrum with only normally distributed noise with variance *σ*^2 ^and mean 0. The baseline should also be a horizontal line at *y *= *b*, so the summation term in the score function $\sum_{i=1}^{n}b_{i}=nb$. The smoothing term $-A\sum_{i}{\left(b_{i+1}+b_{i-1}-2b_{i}\right)}^{2}$ = 0 because the baseline should be horizontal and has no curvature. The expectation value of negativity term could be calculated based on the probability density function (PDF) of the noise *P*(*y*).

(4) $$<-B\sum_{i}{\left(b_{i}-y_{i}\right)}^{2}g(b_{i}-y_{i})>=-nB\int_{-\infty }^{\infty }{\left(b-y\right)}^{2}g(b-y)P(y)dy=-nB\int_{-\infty }^{b}{\left(b-y\right)}^{2}P(y)dy$$

Where $P(y)=\frac{1}{\sqrt{2\pi \sigma^{2}}}\exp(-\frac{y^{2}}{2\sigma^{2}})$ is the PDF of normal distribution with variance *σ*^2 ^and mean 0. The boundary of the integral (-∞, *b*) in equation (4) is determined by explicitly plug in the step function $g(b-y)=\{\begin{array}{cc} 1, & y<b\\ 0, & y\geq b \end{array}$.

Hence the expected value of the score function <*F*(*b*) > becomes:

(5) $$<F(b)>=nb-nB\int_{-\infty }^{b}{\left(b-y\right)}^{2}\cdot \frac{1}{\sqrt{2\pi \sigma^{2}}}\exp(-\frac{y^{2}}{2\sigma^{2}})dy$$

The estimated baseline should be at the zero intensity level, which means the score function reaches its maximum at *b *= 0.

$$\frac{\partial <F>}{\partial b}{|}_{b=0}=n-\frac{2n\sigma B}{\sqrt{2\pi }}=0,$$

This gives us the theoretical value of *B*

(6) $$B=\frac{\sqrt{2\pi }}{2\sigma }\approx \frac{1.25}{\sigma }.$$

So the negativity penalty parameter *B *is determined by the noise standard deviation *σ*. We define the constant $B^{*}=\frac{\sqrt{2\pi }}{2}\approx 1.25$ so that *B *is in the form of $B=\frac{B^{*}}{\sigma }$. By dividing by *σ*, the negativity penalty $-B\sum_{i}{\left(b_{i}-y_{i}\right)}^{2}g(b_{i}-y_{i})$ will have the same order of the intensity as in the first linear summation term $\sum_{i}b_{i}$ in the score function, which guarantees that the maximal point of the score function remain invariant if the spectrum is multiplied by a scalar, so that the corresponding baseline curve will not be affected. For the same reason, the smoothing penalty parameter, denoted by *A *in the score function (2), should also take the form of $A=\frac{CA^{*}}{\sigma }$ to guarantee invariance in spectrum scaling, where A* is a constant and *C *is a coefficient related with the resolution of the spectra and will be discussed later.

For example, if we multiply the spectrum {*x*_*i*_, *y*_*i*_} by a scalar k, we get a new spectrum {*x*_*i*_, *ky*_*i*_}. The noise standard deviation of the scaled spectrum, denoted by *σ*', is also *k *times the original noise standard deviation *σ*: *σ*' = *kσ*.

The score function for the original spectrum is:

(7) $$F(b)=\sum_{i}b_{i}-\frac{CA^{*}}{\sigma }\sum_{i}{\left(b_{i+1}+b_{i-1}-2b_{i}\right)}^{2}-\frac{B^{*}}{\sigma }\sum_{i}{\left(b_{i}-y_{i}\right)}^{2}g(b_{i}-y_{i})$$

And the score function for the scaled spectrum is:

(8) F(b')=∑ibi'−CA∗kσ∑i(bi+1'+bi−1'−2bi')2−B∗kσ∑i(bi'−kyi)2g(bi'−kyi)

The estimated baseline for scaled spectrum should also be also *k *times of the original spectrum baseline, thus **b**' = *k***b**, or equivalently, *b*_*i*_^' ^= *kb*_*i*_. Substitute into equation (8), we have

$$F(b')=\sum_{i}kb_{i}-\frac{CA^{*}}{k\sigma }\sum_{i}{\left(kb_{i+1}+kb_{i-1}-2kb_{i}\right)}^{2}-\frac{B^{*}}{k\sigma }\sum_{i}{\left(kb_{i}-ky_{i}\right)}^{2}g(kb_{i}-ky_{i})=kF(b)$$

So *F*(**b'**) = *F*(*k***b**) = *kF*(**b**), which means the score function is in an invariant form for scaling. Multiplying the spectrum by a constant does not affect finding the optimal baseline by maximizing this score function.

In addition, the smoothness penalty should be robust to the abscissa resolution. For example, if we take half the data points (with odd indices) of the original spectrum so that the chemical shift interval is doubled, the baseline curve should not be affected. This suggests the coefficient *C *in $A=\frac{CA^{*}}{\sigma }$ is related with the abscissa resolution *dx*.

Recall the smoothing term is the sum of squared second order derivatives of {*b*_*i*_}, rewrite it in the generic form of discrete representation of second order derivative:

$$A\sum_{i}{\left(b_{i+1}+b_{i-1}-2b_{i}\right)}^{2}=\frac{A^{*}}{\sigma }\sum_{i}{\left(\frac{b_{i+1}+b_{i-1}-2b_{i}}{dx^{2}}\right)}^{2}=\frac{A^{*}}{\sigma \cdot dx^{4}}\sum_{i}{\left(b_{i+1}+b_{i-1}-2b_{i}\right)}^{2}$$

Therefore *C *has an inverse quadruple relation with the resolution *dx *of the abscissa. For a given spectrum, *dx *is inverse proportional to the number of data points *n*: $dx\propto \frac{1}{n}$, which means *C *could take the value of *n*^4^.

(9) $$\text{So }A=\frac{CA^{*}}{\sigma }=\frac{n^{4}A^{*}}{\sigma },$$

where A* is a constant that is independent of the spectrum. By comparing different spectra, we choose an empirically reasonable value of A* to be A* = 5 × 10^-9^.

Based on the above analysis, the score function takes the follow form

(10) $$F(b)=\sum_{i}b_{i}-\frac{n^{4}A^{*}}{\sigma }\sum_{i}{\left(b_{i+1}+b_{i-1}-2b_{i}\right)}^{2}-\frac{B^{*}}{\sigma }\sum_{i}{\left(b_{i}-y_{i}\right)}^{2}g(b_{i}-y_{i})$$

where A* = 5 × 10^-9^, B* ≈ 1.25, *σ *is the standard deviation of noise and *n *is the total number of data points. The baseline curve is insensitive to small changes of *A *and *B*, unless the orders of magnitude are changed.

The estimation of the noise standard deviation *σ *is based on the model in equation (1), The variance of a certain part of spectrum is derived as the following

(11) $$\begin{array}{ccc} Var(y)=\sigma_{\varepsilon }^{2}+\mu^{2}S^{2}, & \text{where} & S^{2}=\exp(\sigma_{\eta }^{2})[\exp(\sigma_{\eta }^{2})-1] \end{array}$$

**Figure 1:**
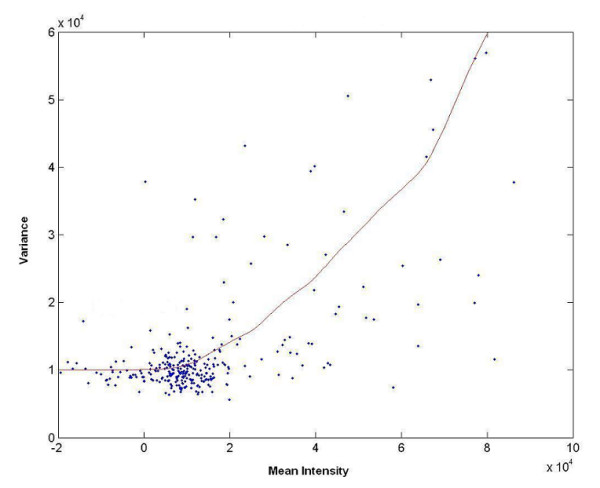
**LOWESS smoothing for variances estimation**. Variances versus mean intensities sampled bins of 1D NMR metabolomics spectra. The bin size was set to 32 data points, corresponding to 0.012 ppm in chemical shift. The fitted LOWESS regression curve was plotted in red, and the flat region of the LOWESS curve represents the estimated noise variance.

It indicates that the signal variance *Var*(*y*) increases with the mean value of signal intensity *μ*. We can estimate the noise variance $\sigma_{\varepsilon }^{2}$ by fitting equation (11) on the signal variances and mean intensities sampled from the spectrum. We divide the spectrum into small regions and compute the variance and mean intensity within each regions. Figure 1 plots the variances versus mean values with region size of 32 data points, corresponding to 0.012 ppm in chemical shifts. We use LOWESS (locally weighted scatterplot smoothing) regression to fit equation (11). The red line in Figure 1 represents the fitted regression line. It has a quadratic form as expressed in equation (11).

Since $Var(y)=\sigma_{\varepsilon }^{2}+\mu^{2}S^{2}\approx \sigma_{\varepsilon }^{2}$ when *μ *≈ 0, the noise variance $\sigma_{\varepsilon }^{2}$ is approximately equal to the signal variance *Var*(*y*) for small mean values. We take the predicted value of *Var*(*y*) at zero mean intensity in the LOWESS regression to be our estimate of $\sigma_{\varepsilon }^{2}$, and the standard deviation of the noise *σ *is the square root of the noise variance

$$\sigma =\sqrt{\sigma_{\varepsilon }^{2}}$$

After determining the parameters, we maximize the function *F*(**b**) to find the baseline **b**_0_, according to equation (3). Mathematically, we solve $\frac{\partial F(b)}{\partial b}=0$ to find the maximum of *F*(**b**). This partial derivative equation expands as a linear system with the solution to be **b**_0_. The numerical implementation of solving this linear system is attached in the appendix.

## Results and discussion

Based on this penalized smoothing model, we test the baseline correction method on simple 1D NMR spectra and complex metabolomics spectra.

**Figure 2:**
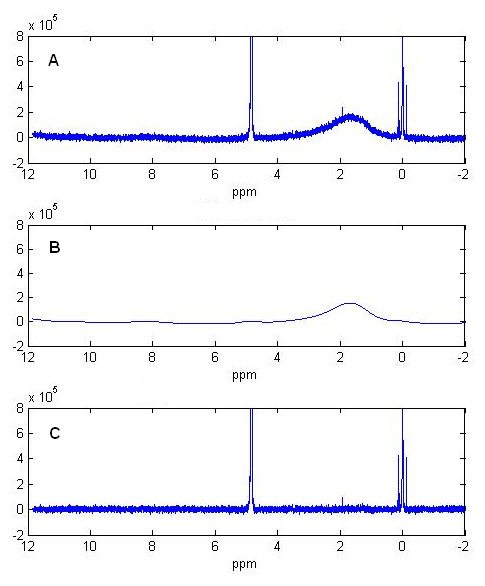
**Baseline correction by penalized parametric smoothing method**. (A) Original 1D proton NMR spectrum of DSS reference with distorted baseline. (B) Detected baseline curve by penalized parametric smoothing method. (C) Corrected spectrum after baseline subtraction.

Figure 2 demonstrates this method corrects the baseline distortion of a simple 1D ^1^H NMR reference spectrum of DSS (2,2-Dimethyl-2-silapentane-5-sulfonic acid) with *n *= 65536 data points. The estimated noise standard deviation is *σ *= 8335.9. According to previous discussion of parameter determination, the parameters are set to $A=\frac{n^{4}A^{*}}{\sigma }=1.1\times 10^{7}$ and $B=\frac{B^{*}}{\sigma }=1.5\times 10^{-4}$. Figure 2A shows the original spectrum with apparent baseline distortions. This distorted baseline is detected by the penalized smoothing method in Figure 2B. In Figure 2C, this baseline curve is subtracted from the spectrum and the distortion is corrected. The optimal baseline found by our baseline model fits well with the distortion curve. The small peak at 2ppm in the spectrum is correctly presented after baseline subtraction.

**Figure 3:**
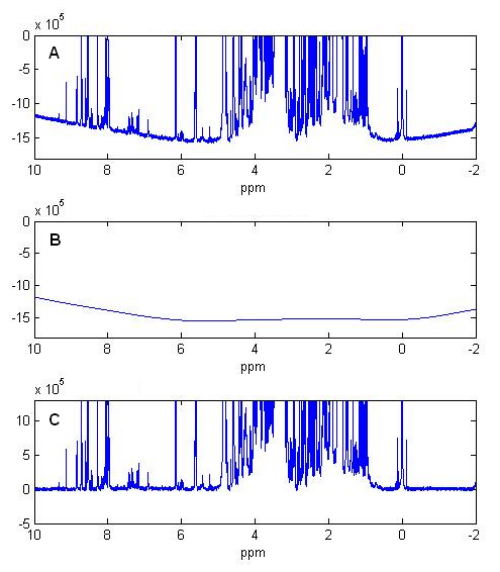
**Baseline correction for metabolomics spectrum by penalized parametric smoothing method**. (A) Original spectrum with distorted baseline. (B) Detected baseline curve by penalized parametric smoothing method. (C) Corrected spectrum after baseline subtraction.

We test this method in more complicated metabolomics spectra collected from tissue samples of red abalone. The data are from a study of environmental stresses on the development of a bacterial infection among red abalones (*Haliotis rufescens*) [12,13]. The dataset include 65 1D proton NMR spectra with 65536 data points in each spectrum. In our test the penalized smoothing method correctly detected and removed the distorted baseline for all 65 spectra. Figure 3 shows the baseline correction result on one example of testing spectra using the penalized smoothing method. In Figure 3A, the peaks of metabolites aggregate together and form continuous peak regions. Lack of noise points in these regions generates big gaps in baseline construction. As demonstrated in Figure 3B and Figure 3C, the baseline distortion is correctly detected and removed. In these gaps, the baseline curve is constructed following the smoothing constraints in the penalized smoothing model. The penalized smoothing method does not require explicit identification of noise region identification and therefore avoids constructing the baseline by interpolation, which is sensitive to the identification of noise points, especially in the region with high peak densities where noise points is difficult to detect accurately.

**Figure 4:**
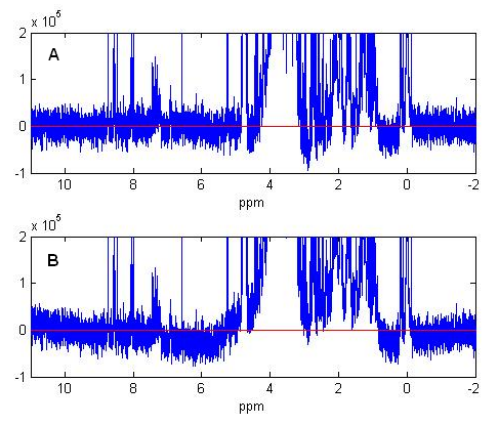
**Comparison of penalized smoothing method and XWIN-NMR 3.5**. (A) Spectrum corrected by penalized parametric smoothing method. (B) Spectrum corrected by automatic baseline correction function in XWIN-NMR 3.5. The red lines represent the zero intensity level.

**Figure 5:**
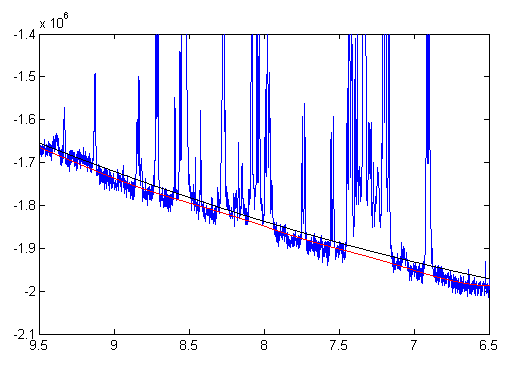
**Comparison of baseline distortion detection by penalized smoothing methods and XWIN-NMR 3.5**. This is an expanded region of the spectrum in Figure 4 before baseline correction. Baseline curves are detected by penalized parametric method (red) and XWIN 3.5 automatic baseline correction function (black).

We compared the penalized smoothing method with commercial software in Figure 4 and Figure 5. We applied the penalized smoothing method and the automatic baseline correction function in XWIN-NMR software (version 3.5) carried by the Bruker AVANCE 600 NMR facility to the same spectrum with baseline distortion, and plot the baseline corrected spectrum in Figure 4. The red lines represent the ideal horizontal baseline at zero intensity level. Both methods are capable of removing large distortions of the baseline and setting the corrected baseline to near the zero intensity level. The corrected baseline by the penalized smoothing method (Figure 4A) fits well with the ideal horizontal baseline in red. In Figure 4B, the corrected baseline by XWINNMR has apparently larger deviation from the zero intensity level.

In Figure 5 we show a region from 6.5 ppm to 9.5 ppm of original spectrum to illustrate the difference of baselines detected by penalized smoothing methods (in red) and XWINNMR 3.5 (in black). The penalized smoothing baseline is closer to the center of noise regions and therefore detects the offsets of signals more accurately. This leads to a more accurate identification and quantification of signals in the corrected spectrum, especially for the small peaks at 7.08 ppm, 8.35 ppm and 8.94 ppm in Figure 5. For all 65 testing spectra, the penalized smoothing method shows significant improvement in presenting low intensity peaks in 47 spectra, corresponding to a percentage of 69%. To achieve comparable baseline correction quality in these spectra, XWINNMR 3.5 needs manually marking of the noise points or regions for baseline interpolation, which is time consuming for batch processing and is dependent on the experience of the software users. The penalized smoothing method is fully automatic and user independent.

## Conclusion

We propose an alternative baseline correction method based on a penalized smoothing model. This method constructs the baseline by maximizing a score function (eq 2) that evaluates how well the baseline fits the spectrum. The model parameters A and B in score function are determined by the noise variance of the spectrum, *σ*^2^, which can be automatically estimated by LOWESS regression. This method does not require explicit identification of noise data points for baseline interpolation, or assumption of fixed forms of baseline curves, i.e. polynomials etc. Instead, it uses a general smoothing term to fit flexible forms of baseline distortion.

We applied this method to 1D NMR spectra with baseline distortion, and demonstrate it is effective for both regular 1D NMR spectra and metabolomics spectra with over-crowded peaks. The numerical implementation is fast and stable on common personal computers. This method provides an alternative to the existing noise detection and interpolation approaches in baseline correction, especially for spectra with many crowded peaks, such as in NMR metabolomics profiling, where noise points are more difficult to identify accurately. Compared with the widely used XWINNMR software, the method provided more accurate baseline correction on 47 out of 65 of our testing metabolomics spectra. With a few modifications, this penalized smoothing baseline correction approach is also applicable to 2D NMR spectra. The numerical implementation and optimization for 2D baseline correction could be one topic in further study.

## Appendix

We solve $\frac{\partial F}{\partial b}=0$ to find the maximum of score function *F*(**b**), which leads to linear system of order *n*. (*n *is the number of data points in **b**).

**Db **= **m**

**D **is the Hessian matrix of *F*(**b**), i.e.$D_{ij}=\frac{\partial^{2}F}{\partial b_{i}\partial b_{j}}$, and **m **is a vector. In detail, the entries of **D **and **m **have the following entries:

$$\begin{array}{l} \begin{array}{cc} m_{i}=\{\begin{array}{c} 2By_{i}-1,\\ -1 \end{array} & \begin{array}{c} b_{i}>y_{i}\\ b_{i}\leq y_{i} \end{array}; \end{array}\\ \begin{array}{cc} D_{ii}=\frac{\partial^{2}F}{\partial b_{i}^{2}}=\{\begin{array}{c} 12A+2B,\\ 12A, \end{array} & \begin{array}{c} b_{i}>y_{i}\\ b_{i}\leq y_{i} \end{array}; \end{array}\\ \begin{array}{cc} D_{i,i\pm 1}=D_{i\pm 1,i}=\frac{\partial^{2}F}{\partial b_{i}\partial b_{i\pm 1}}=-8A; & D_{i,i\pm 2}=D_{i\pm 2,i}=\frac{\partial^{2}F}{\partial b_{i}\partial b_{i\pm 2}}=2A. \end{array} \end{array}$$

The boundary points may lack the neighbor points in the above formulas, and need to be treated separately. The non-existent terms in these equations are omitted:

$$\begin{array}{l} \begin{array}{cc} D_{11}=D_{nn}=\{\begin{array}{c} 2A+2B,\\ 2A, \end{array} & \begin{array}{c} b_{i}>y_{i}\\ b_{i}\leq y_{i} \end{array}; \end{array}\\ \begin{array}{cc} D_{22}=D_{n-1,n-1}=\{\begin{array}{c} 10A+2B,\\ 10A, \end{array} & \begin{array}{c} b_{i}>y_{i}\\ b_{i}\leq y_{i} \end{array}; \end{array}\\ D_{12}=D_{21}=D_{n,n-1}=D_{n-1,n}=-4A. \end{array}$$

We use an iterated procedure to solve **Db **= **m **because the entries of **D **and **m **are dependent on **b**, and need to be updated in each iteration until it converges. This procedure is described in the following steps:

1) Set the initial value of baseline points **b **to be zeros.

2) determine **D **and **m **based on current values of **b**

3) solve **Db **= **m **for **b**

4) stop if the relative change of **b **is smaller than a threshold, otherwise go to step 2)

The Hessian matrix **D **is highly sparse, with all the non-zero elements in a symmetric band along the main diagonal. This kind of matrix structure can be solved very efficiently. In addition, the matrix is positive semi-definite, which guarantees the convergence of the iteration, so it is not sensitive to the initial value of **b**. We use MATLAB to implement the above algorithm and the code is available upon request.
